# *Alternaria* species in section *Alternaria* associated with *Iris* plants in China

**DOI:** 10.3389/fmicb.2022.1036950

**Published:** 2022-10-21

**Authors:** Ya-Nan Gou, Sein Lai Lai Aung, Aye Aye Htun, Cheng-Xin Huang, Jian-Xin Deng

**Affiliations:** ^1^Department of Plant Protection, College of Agriculture, Yangtze University, Jingzhou, China; ^2^National Key Laboratory of Wheat and Maize Crop Science, Agronomy College, Henan Agricultural University, Zhengzhou, China

**Keywords:** *Alternaria*, *Iris*, morphology, phylogenetic analysis, pathogenicity

## Abstract

Plants of the *Iris* genus have been widely cultivated because of their medicinal, ornamental, and economic values. It commonly suffers from *Alternaria* leaf spot or blight disease leading to considerable losses for their commercial values. During an investigation of 14 provinces or municipalities of China from 2014 to 2022, a total of 122 *Alternaria* strains in section *Alternaria* were obtained from diseased leaves of *Iris* spp.. Among them, 12 representative strains were selected and identified based on morphological characterization and multi-locus phylogenetic analysis, which encompassed the internal transcribed spacer of rDNA region (ITS), glyceraldehyde-3-phosphate dehydrogenase (*GAPDH*), translation elongation factor 1 alpha (*TEF1*), RNA polymerase second largest subunit (*RPB2*), *Alternaria* major allergen gene (*Alt a 1*), an anonymous gene region (OPA10-2), and endopolygalacturonase gene (*EndoPG*). The strains comprised two known species of *A. alternata* and *A. iridicola*, and two new species of *A. setosae* and *A. tectorum*, which were described and illustrated here. Their pathogenicity evaluated on *Iris setosa* indicated that all the strains could induce typical *Alternaria* leaf spot or blight symptoms. The results showed that the virulence was variable among those four species, from which *A. tectorum* sp. nov. was the most virulent one, followed by *A. setosae* sp. nov., *A. iridicola* and *A. alternata*.

## Introduction

The *Iris* Linn. is the largest genus in the Iridaceae containing about 300 species in worldwide (Xiang et al., 2020). It is one of the most extensively cultivated plants in modern landscaping because it is a great ground cover material for urban greening (Zhao et al., 2000; Clair, 2005; Li et al., 2020). In China, the plants have been widely grown as a year-round ornamental with various large and colorful flowers (Yang et al., 2017). Some *Iris* species are rich in beneficial chemicals, served as effective pharmaceutical ingredient to treat various diseases including cancer, inflammation, bacterial and viral infections (Tahara et al., 1991; Huang, 2004; Meng et al., 2016). In addition, *Iris* plants are also utilized in the perfume and cosmetic industries considering the fragrance reasonability (Wang et al., 2010).

During the cultivation of *Iris* spp., the plants suffer from various diseases induced by fungal, bacterial, viral, and nematode pathogens (Umesha et al., 2010; Lu et al., 2016; Wang et al., 2020; Wang et al., 2022). Pathogenic fungal species have been commonly found in connection with the genera of *Alternaria*, *Botrytis*, *Colletotrichum*, *Fusarium*, *Harzia* and *Puccinia* (Bobev, 2009; Liu et al., 2016; Schultes et al., 2017; Choi et al., 2019; Wang et al., 2022). Leaf spot and blight diseases caused by *Alternaria* species in sect. *Alternaria* is prevalent on *Iris* plants worldwide presenting the typical symptoms of brown spot with a yellow halo or blighted leaf, which greatly reduce their ornamental values. *Alternaria iridicola* in China, Japan, and USA (Zhang, 2003; Simmons, 2007; Nishikawa and Nakashima, 2020), *A. iridiaustralis* in Australia, China, and New Zealand (Simmons, 2007; Luo et al., 2018), *A. tenuissima* in China (Zhang, 2003; Sun et al., 2019; Li et al., 2020) have been reported as fungal pathogens on *Iris* plants.

The *Alternaria* is a ubiquitous fungus comprising many destructive plant pathogens, which can result in economic losses on a large number of significant agronomic crops and ornamentals (Thomma, 2003; Kahl et al., 2015). There has been a longtime controversy on the classification criteria of *Alternaria* (Lawrence et al., 2016). Simmons proposed a reasonable standard for the morphological taxonomy of *Alternaria* species based on sporulation patterns and conidial morphology, by which around 280 species were described and summarized into two sections of large-spored and small-spored of *Alternaria* (Simmons, 2007). Since the 20th century, molecular taxonomic structure has been developed and applied to identify *Alternaria* species with various gene fragments (Pryor and Gilbertson, 2000; Hong et al., 2005; Lawrence et al., 2012, 2013; Woudenberg et al., 2013, 2014; He et al., 2021). Recently, the multi-locus phylogenetic analysis plays an important role in assisting *Alternaria* classification (Woudenberg et al., 2015; Zheng et al., 2015; Sofie et al., 2017; Cheng et al., 2022). It separates *Alternaria* into 29 sections and 7 monotypic lineages (Woudenberg et al., 2013; Marin-Felix et al., 2019; Bessadat et al., 2020; Gannibal et al., 2022; Huang et al., 2022). Besides, in combination with morphology and molecular approaches is commonly used to determine the *Alternaria* up to species levels (Lawrence et al., 2016; Gannibal, 2019; Bessadat et al., 2021; Zhao et al., 2022).

One of the large *Alternaria* sections, sect. *Alternaria* contains most of the small-spored *Alternaria* species with concatenated conidia including about 60 morphological or host-specific species (Simmons, 2007; Woudenberg et al., 2015), which are frequently encountered on the disease leaf samples of *Iris* plants. To know the species population associated with *Iris* plants in China, a large-scale sample collection and fungal isolation had been conducted from 2014 to 2022, from which a total of 122 strains of sect. *Alternaria* were obtained. To comprehend their species levels, this study aims to identify the species using morphological traits and multi-locus phylogenetic analysis. In addition, their variety of virulence is assessed on *Iris setosa*.

## Materials and methods

### Sampling and isolation

**Figure 1:**
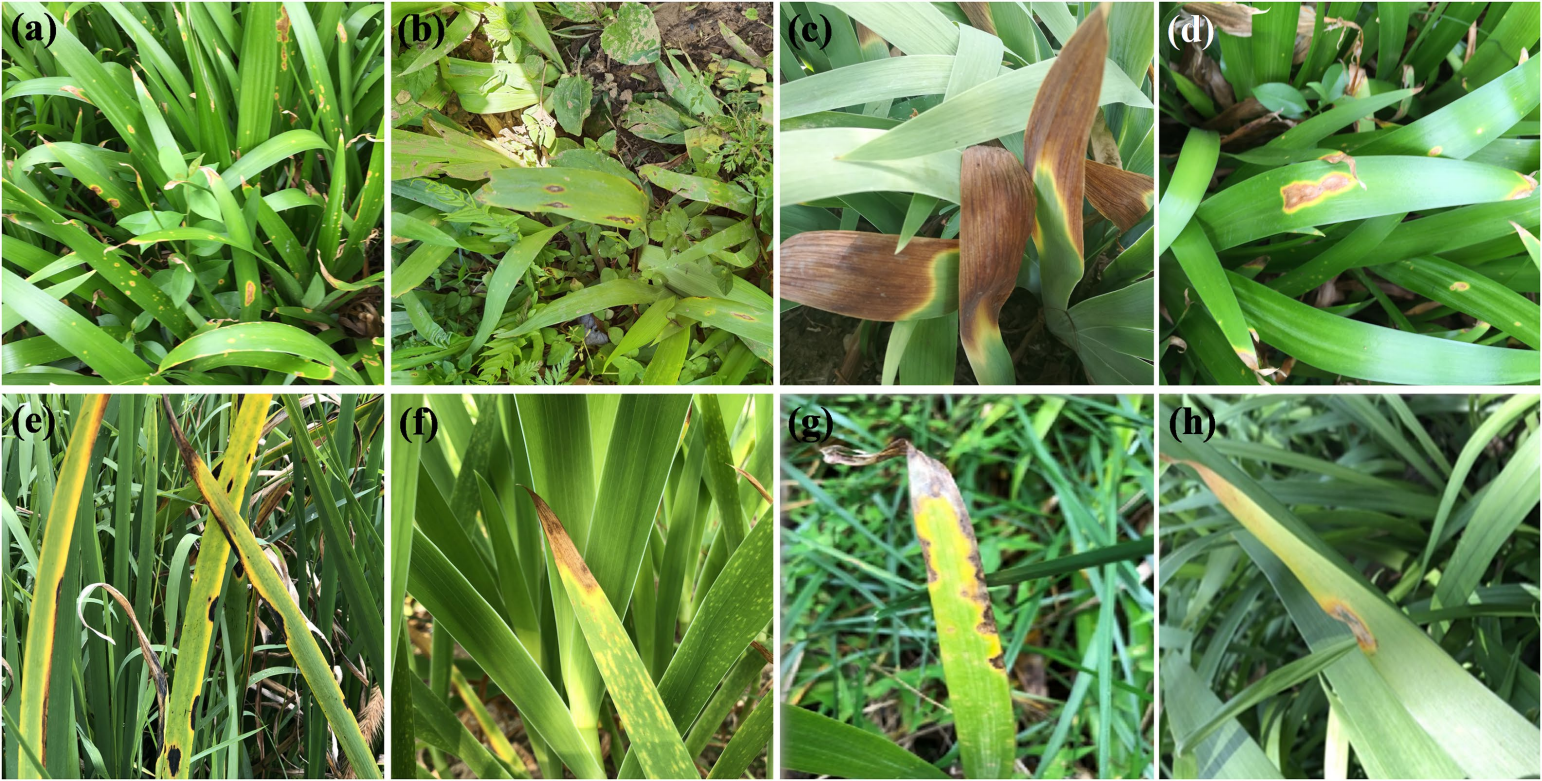
Leaf spot and blight symptoms of *Iris* plants caused by *Alternaria* spp. in field.

During a large-scale investigation on *Alternaria* species in sect. *Alternaria* associated with *Iris* spp. in China (Figure 1), diseased leaf samples were collected from 14 provinces or municipalities (Table 1). For the isolation of *Alternaria*, the samples were cut into small pieces, placed on moist filter papers in Petri dishes and incubated at 25°C for the sporulation (Liu et al., 2019). The samples were observed using a stereomicroscope. The single spore was picked by a sterile glass needle and inoculated onto potato dextrose agar (PDA: Difco, Montreal, Canada). A total of 122 strains were obtained and kept into test-tube slants stored at 4°C in the Fungal Herbarium of Yangtze University (YZU), Jingzhou, Hubei, China. Twelve representative strains (Table 2) representing different species were selected for the present study after pre-morphological, pre-phylogenetic, and pre-pathogenicity assays.

**Table 1:** Location and number of *Alternaria* strains collected from diseased *Iris* leaves in China.

**Host**	**Total strains**	**Location**	
		**Province**	**City**
*Iris hexagona*	30	Jiangsu	Nanjing
		Guangxi	Nanning
		Shanghai	Shanghai
		Yunnan	Kuumainn
*Iris tectorum*	67	Hubei	Jingzhou, Yichang
		Beijing	Beijing
		Shanxi	Taigu, Yangquan
		Shaanxi	Xi’an
		Gansu	Linxia, Lanzhou
		Jiangsu	Nanjing
		Sichuan	Deyang
		Henan	Henan
		Fujian	Fuzhou
*Iris japonica*	18	Fujian	Fuzhou
		Jiangxi	Lushan
		Shannxi	Xi’an
*Iris ensata*	7	Jilin	Changchun
		Gansu	Linxia
		Yunnan	Xisun

**Table 2:** *Alternaria* strains used in this study and the GenBank accession numbers.

**Species/Strain**	**Host/Substrate**	**Country**	**GenBank accession number**						
			**ITS**	**GAPDH**	**TEF1**	**RPB2**	**Alt a 1**	**EndoPG**	**OPA10-2**
***A. alstroemeriae***									
CBS 118808 R	*Alstroemeria* sp.	Australia	KP124296	KP124153	KP125071	KP124764	KP123845	KP123993	KP124601
CBS 118809 T	*Alstroemeria* sp.	Australia	KP124297	KP124154	KP125072	KP124765	KP123845	KP123994	KP124602
***A. alternantherae***									
CBS 124392	*Solanum melongena*	China	KC584179	KC584096	KC584633	KC584374	KP123846	–	–
***A. alternata***									
CBS 916.96 T	*Arachis hypogaea*	India	AF347031	AY278808	KC584634	KC584375	AY563301	JQ811978	KP124632
CBS 102604	*Minneola tangelo*	Israel	KP124334	AY562410	KP125110	KP124802	AY563305	KP124035	KP124643
CBS 102602	*Minneola tangelo*	Turkey	KP124332	KP124187	KP125108	KP124800	KP123881	AY295023	KP124641
CBS 102599	*Minneola tangelo*	Turkey	KP124330	KP124185	KP125106	KP124798	KP123879	KP124032	KP124639
CBS 102595	*Citrus jambhiri*	USA	FJ266476	AY562411	KC584666	KC584408	AY563306	KP124029	KP124636
CBS 127672	*Astragalus bisulcatus*	USA	KP124382	KP124234	KP125160	KP124852	KP123930	KP124086	KP124695
CBS 103.33	Soil	Egypt	KP124302	KP124159	KP125077	KP124770	KP123852	KP123999	KP124607
CBS 102.47	*Citrus sinensis*	USA	KP124303	KP124160	KP125079	KP124772	KP123854	KP124001	KP124609
CBS 117.44	*Godetia* sp.	Denmark	KP124303	KP124160	KP125079	KP124772	KP123854	KP124001	KP124609
CBS 102596	*Citrus jambhiri*	USA	KP124328	KP124183	KP125104	KP124796	KP123877	KP124030	KP124637
CBS 918.96	*Dianthus chinensis*	UK	AF347032	AY278809	KC584693	KC584435	AY563302	KP124026	KP124633
CBS 121455	*Broussonetia papyrifera*	China	KP124368	KP124220	KP125146	KP124838	KP123916	KP124072	KP124681
CBS 121547	*Pyrus bretschneideri*	China	KP124372	KP124224	KP125150	KP124842	KP123920	KP124076	KP124685
CBS 127671	*Stanleya pinnata*	USA	KP124381	KP124233	KP125159	KP124851	KP123929	KP124085	KP124694
CBS 106.34	*Linum usitatissimum*	Unknown	Y17071	JQ646308	KP125078	KP124771	KP123853	KP124000	KP124608
CBS 121336	*Allium* sp.	USA	KJ862254	KJ862255	KP125141	KP124833	KJ862259	KP124067	KP124676
CBS 119543	*Citrus paradisi*	USA	KP124363	KP124215	KP125139	KP124831	KP123911	KP124065	KP124674
CBS 195.86	*Euphorbia esula*	Canada	KP124317	KP124173	KP125093	KP124785	JQ646398	KP124017	KP124624
CBS 119399	*Minneola tangelo*	USA	KP124361	JQ646328	KP125137	KP124829	KP123910	KP124063	KP124672
CBS 479.90	*Citrus unshiu*	Japan	KP124319	KP124174	KP125095	KP124787	KP123870	KP124019	KP124626
CBS 121348	*Platycodon grandiflorus*	China	KP124367	KP124219	KP125144	KP124836	KP123915	KP124070	KP124679
CBS 595.93	*Pyrus pyrifolia*	Japan	KP124320	KP124175	KP125096	KP124788	JQ646399	KP124020	KP124627
CBS 118818	*Vaccinium* sp.	USA	KP124359	KP124213	KP125135	KP124827	KP123908	KP124061	KP124671
CBS 118814	*Solanum lycopersicum*	USA	KP124357	KP124211	KP125133	KP124825	KP123906	KP124059	KP124669
CBS 118815	*Solanum lycopersicum*	USA	KP124358	KP124212	KP125134	KP124826	KP123907	KP124060	KP124670
CBS 118811	*Brassica oleracea*	USA	KP124356	KP124210	KP125132	KP124824	KP123904	KP124057	KP124667
CBS 118812	*Daucus carota*	USA	KC584193	KC584112	KC584652	KC584393	KP123905	KP124058	KP124668
CBS 119408	*Euphorbia esula*	USA	KP124362	JQ646326	KP125138	KP124830	JQ646410	KP124064	KP124673
CBS 540.94	*Nicotiana tabacum*	USA	AY278835	AY278811	KC584667	KC584409	AY563304	KP124147	KP124758
CBS 121333	*Nicotiana tabacum*	USA	KP124444	KP124293	KP125223	KP124914	KP123990	KP124150	KP124761
CBS 104.32	*Gossypium* sp.	Zimbabwe	KP124430	JQ646312	KP125209	KP124900	JQ646395	KP124135	KP124746
CBS 102601	*Minneola tangelo*	Colombia	KP124433	KP124282	KP125212	KP124903	KP123979	KP124138	KP124749
CBS 102597	*Minneola tangelo*	USA	KP124432	KP124281	KP125211	KP124902	KP123978	KP124137	KP124748
**YZU 171270**	***Iris tectorum***	China	OP341610	OP352298	OP374451	OP352286	OP293709	OP293721	OP352274
**YZU 171499**	***Iris tectorum***	China	OP341534	OP352295	OP374448	OP352283	OP293708	OP293719	OP352271
**YZU 181050**	***Iris tectorum***	China	OP341604	OP352297	OP374450	OP352285	OP293707	OP293720	OP352273
**YZU 181280**	***Iris tectorum***	China	OP341598	OP352296	OP374449	OP352284	OP293706	OP293718	OP352272
***A. arborescens***									
CBS 101.13	Peat soil	Switzerland	KP124392	KP124244	KP125170	KP124862	KP123940	KP124096	KP124705
CBS 119545	*Senecio skirrhodon*	New Zealand	KP124409	KP124260	KP125187	KP124879	KP123956	KP124113	KP124723
CBS 119544	*Avena sativa*	New Zealand	KP124408	JQ646321	KP125186	KP124878	KP123955	KP124112	KP124722
CBS 112749	*Malus domestica*	South Africa	KP124402	KP124254	KP125180	KP124872	KP123949	KP124106	KP124716
CBS 105.24	*Solanum tuberosum*	Unknown	KP124393	KP124245	KP125171	KP124863	KP123941	KP124097	KP124706
CBS 126.60	Wood	UK	KP124397	KP124249	KP125175	KP124867	JQ646390	KP124101	KP124710
CBS 109730	*Solanum lycopersicum*	USA	KP124399	KP124251	KP125177	KP124869	KP123946	KP124103	KP124713
CBS 105.49	Contaminant blood culture	Italy	KP124396	KP124248	KP125174	KP124866	KP123944	KP124100	KP124709
***A. betae-kenyensis***									
CBS 118810	*Beta vulgaris* var. *cicla*	Kenya	KP124419	KP124270	KP125197	KP124888	KP123966	KP124123	KP124733
***A. burnsii***									
CBS 130264	*Human sputum*	India	KP124425	KP124275	KP125203	KP124894	KP123972	KP124129	KP124739
CBS 108.27	*Gomphrena globosa*	Unknown	KC584236	KC584162	KC584727	KC584468	KP123850	KP123997	KP124605
CBS 107.38 T	*Cuminum cyminum*	India	KP124420	JQ646305	KP125198	KP124889	KP123967	KP124124	KP124734
CBS 118816	*Rhizophora mucronata*	India	KP124423	KP124273	KP125201	KP124892	KP123970	KP124127	KP124737
CBS 118817	*Tinospora cordifolia*	India	KP124424	KP124274	KP125202	KP124893	KP123971	KP124128	KP124738
***A. citricancri***									
CBS 119543 T	*Citrus paradisi*	USA	KP124363	KP124215	KP125139	KP124831	KP123911	KP124065	KP124674
***A. eichhorniae***									
CBS 489.92 T	*Eichhornia crassipes*	India	KC146356	KP124276	KP125204	KP124895	KP123973	KP124130	KP124740
CBS 119778 R	*Eichhornia crassipes*	Indonesia	KP124426	KP124277	KP125205	KP124896	KP123973	KP124131	KP124741
***A. gaisen***									
CBS 632.93 R	*Pyrus pyrifolia*	Japan	KC584197	KC584116	KC584658	KC584399	KP123974	AY295033	KP124742
CBS 118488 R	*Pyrus pyrifolia*	Japan	KP124427	KP124278	KP125206	KP124897	KP123975	KP124132	KP124743
CPC 25268	Unknown	Portugal	KP124428	KP124133	KP125207	KP124279	KP123976	KP124898	KP124744
***A. gossypina***									
CBS 102597	*Minneola tangelo*	USA	KP124432	KP124281	KP125211	KP124902	KP123978	KP124137	KP124748
CBS 104.32 T	*Gossypium* sp.	Zimbabwe	KP124430	KP124135	KP125209	JQ646312	JQ646395	KP124900	KP124746
CBS 102601	*Minneola tangelo*	Colombia	KP124433	KP124282	KP125212	KP124903	KP123979	KP124138	KP124749
***A. iridiaustralis***									
CBS 118404 R	*Iris* sp.	New Zealand	KP124434	KP124283	KP125213	KP124904	KP123980	KP124139	KP124750
CBS 118486 T	*Iris* sp.	Australia	KP124435	KP124284	KP125214	KP124905	KP123981	KP124140	KP124751
***A. iridicola***									
MUCC 2149 T	*Iris japonica*	Japan	LC269975	LC270143	LC275061	LC275241	LC276239	LC276254	–
**YZU 161051**	*Iris tectorum*	China	OP341633	OP352302	OP374455	OP352290	OP293713	OP293725	OP352278
**YZU 161119**	*Iris tectorum*	China	OP341632	OP352301	OP374454	OP352289	OP293712	OP293724	OP352277
**YZU 161186**	*Iris tectorum*	China	OP341622	OP352300	OP374453	OP352288	OP293711	OP293723	OP352276
**YZU 191090**	*Iris japonica*	China	OP341617	OP352299	OP374452	OP352287	OP293710	OP293722	OP352275
***A. jacinthicola***									
CBS 133751 T	*Eichhornia crassipes*	Mali	KP124438	KP124287	KP125217	KP124908	KP123984	KP124143	KP124754
CBS 878.95	*Arachis hypogaea*	Mauritius	KP124437	KP124286	KP125216	KP124907	KP123983	KP124142	KP124753
***A. longipes***									
CBS 540.94	*Nicotiana tabacum*	USA	AY278835	KP124147	KC584667	AY278811	AY563304	KC584409	KP124758
CBS 121333 R	*Nicotiana tabacum*	USA	KP124444	KP124150	KP125223	KP124293	KP123990	KP124914	KP124761
***A. tomato***									
CBS 114.35	*Solanum lycopersicum*	Unknown	KP124446	KP124295	KP125225	KP124916	KP123992	KP124152	KP124763
CBS 103.30	*Solanum lycopersicum*	Unknown	KP124445	KP124294	KP125224	KP124915	KP123991	KP124151	KP124762
***A. tectorum***									
**YZU 161050 T**	*Iris tectorum*	China	OP341728	OP352303	OP374456	OP352291	OP293714	OP293726	OP352281
**YZU 161052**	*Iris tectorum*	China	OP341817	OP352304	OP374457	OP352292	OP293715	OP293727	OP352282
***A. setosa***									
**YZU 191076**	*Iris japonica*	China	OP341739	OP352305	OP374458	OP352293	OP293716	OP293728	OP352279
**YZU 191101 T**	*Iris japonica*	China	OP2341770	OP352306	OP374459	OP352294	OP293717	OP293729	OP352280

The present *Alternaria* strains of this study are marked in bold; T: ex-type isolate, R: representative isolate.

### Morphology

The *Alternaria* strains were cultured on PDA at 25°C for 7 days in darkness to determine the cultural features. To examine the conidial morphology, fresh mycelia were grown on potato carrot agar (PCA) and V8 juice agar (V8A) media, then incubated at 22°C with a light period of 8 h light/16 h dark (Simmons, 2007). After 7 days, morphological characteristics of sporulation patterns, conidiophores and conidia were visualized and photographed with a Nikon Eclipse Ni-U microscope system (Nikon, Japan). Fifty randomly selected conidia for each strain were observed and measured to determine the morphology.

### DNA extraction and PCR amplification

Genomic DNA was extracted from mycelium scraped from the surface of 5-day-old colonies on PDA using the CTAB method described in Watanabe et al. (2010). Seven gene regions including internal transcribed spacer of rDNA region (ITS), glyceraldehyde-3-phosphate dehydrogenase (*GAPDH*), translation elongation factor 1 alpha (*TEF1*), RNA polymerase second largest subunit (*RPB2*), *Alternaria* major allergen gene (*Alt a 1*), an anonymous gene region (OPA10-2), and endopolygalacturonase (*EndoPG*) were used for phylogenic analysis. The PCR amplifications were performed with primer pairs of ITS5/ITS4 (White et al., 1990), gpd1/gpd2 (Berbee et al., 1999), EF1-728F/EF1-986R (Carbone and Kohn, 1999), RPB2-5F/RPB2-7cR (Liu et al., 1999), Alt-for/Alt-rev (Hong et al., 2005), OPA10-2 l/OPA10-2R (Andrew et al., 2009) and PG3/PG2b (Andrew et al., 2009), respectively. A 25-μL PCR mixture contained 21 μl 1.1× *Taq* PCR Star Mix (TSINGKE, Beijing, China), 2 μl template DNA, and 1 μl of each primer was performed in a BIO-RAD T100 thermo cycler. The amplified program for PCR amplifications of the seven gene regions was referenced from Woudenberg et al. (2015). Successful amplified products were purified and sequenced by TSINGKE company (Beijing, China). The obtained sequences for each gene were deposited in GenBank^1^ with the accession numbers indicated in Table 2.

### Phylogenetic analysis

The ITS, *GAPDH*, *TEF1*, *RPB2*, *Alt a 1*, OPA10-2 and *EndoPG* gene sequences were launched for BLAST searches in NCBI.^2^ Their relevant sequences were retrieved from GenBank database and referenced from Woudenberg et al. (2015; Table 2). *Alternaria alternantherae* (CBS 124392) from *Alternaria* section of *Alternantherae* was used as outgroup taxon (Table 2). Sequences were aligned and edited by the program of PHYDIT v3.2 (Chun, 1995). The seven gene sequences were concatenated and edited manually in MEGA v.7.0.26 (Kumar et al., 2016). Phylogenetic trees were constructed based on Bayesian inference (BI) analysis using MrBayes v.3.1.2 (Ronquist and Huelsenbeck, 2003), and maximum likelihood (ML) method using RAxML v.7.2.8 (Stamatakis, 2006). The best-fit model for the data was calculated by the Akaike Information Criterion (AIC) using MrModelTest v. 2.3 (Posada and Crandall, 1998). The Bayesian analysis (MrBayes v. 3.2.1; Ronquist et al., 2012) of two simultaneous Markov Chain Monte Carlo (MCMC) chains were run from random trees for 10, 000, 000 generations and sampled every 100 th generations. The first 25% of the samples were discarded as the burn-in and the run was automatically stopped when the average standard deviation of split frequencies reached below 0.01. The phylogram was plotted and edited in Figtree v.1.3.1 (Rambaut and Drummond, 2010). The branch support for the ML analysis was assessed with 1,000 replicates.

### Pathogenicity tests

*Iris setosa* was commonly cultivated as an ornamental or medicinal plant in China. The plants were purchased from the local market and transplanted into clean pots with organic sterile soil grown in greenhouse under a light period (12 h) at 25°C for 2 months. The test strains were cultured on PDA at 25°C for 5 days, and a diameter of 6 mm disc was removed from the edge of colonies. It was inoculated on wounded living leaves of *I. setosa* helped with a fine sterile needle (eight punctures). Clean PDA discs were used as controls. To ensure an accuracy of pathogenicity assessment, a consistent treatment method was adopted for each assay. For each strain, two plants were inoculated with four sites, which were replicated for three times. To confirm the Koch’s law, the pathogen was re-isolated from the diseased symptoms and compared with the original strains based on morphology. The disease incidence was recorded, and the lesion size (LS = the maximum lesion length) was measured after 7 days. The LS values were the mean value of three replicates ± standard deviation. The least significant difference test (*p* < 0.05) was analyzed by IBM SPSS Statistics 23.

## Results

### Phylogenetic analysis

**Figure 2:**
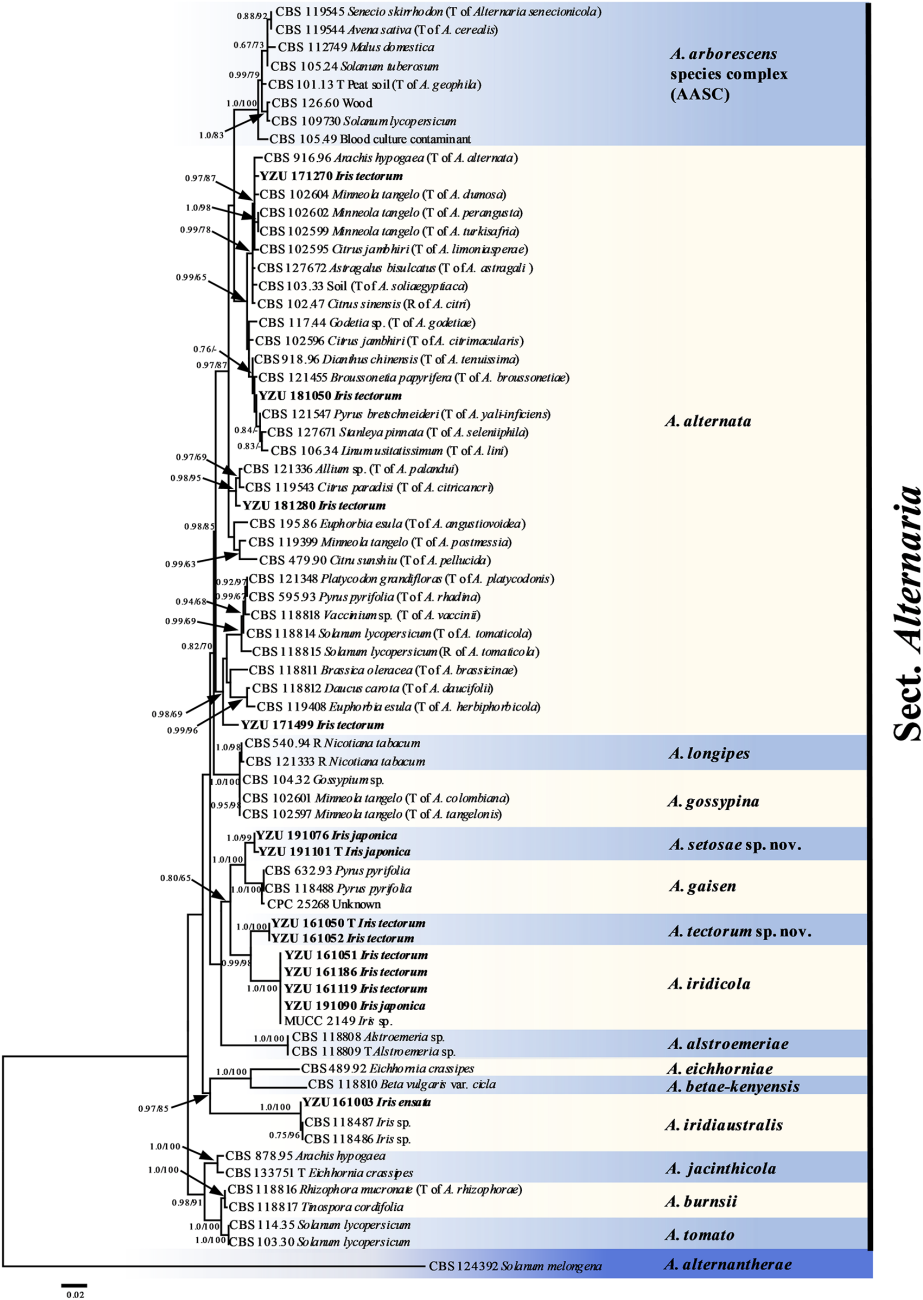
Phylogenetic tree based on the combined gene sequences of ITS, *GAPDH*, *TEF1*, *RPB2*, *Alt a 1*, OPA10-2 L and *EndoPG* generated from *Alternaria* spp. on *Iris* plants. The Bayesian posterior probabilities (PP > 0.60) and maximum likelihood bootstrap values (BS > 60%) are given at the nodes (PP/BS). Examined present strains are in bold.

On the basis of BLAST searches, all 122 *Alternaria* strains were belonged to sect. *Alternaria*. Phylogenetic analyses using the combined dataset of the seven gene sequences (ITS, *GAPDH*, *TEF1*, *RPB2*, *Alt a 1,* OPA10-2 and *EndoPG*) were conducted including 79 strains of sect. *Alternaria* (Table 2), which comprised 3,340 characters (470 from ITS，534 from *GAPDH*，240 from *TEF1*，603 from *RPB2*，429 from *Alt a 1*，622 from OPA10-2，442 from *EndoPG*). The resulted topologies of BI and ML analysis were similar to each other, and the ML tree was shown in Figure 2 for basal phylogeny. It showed that strains of YZU 171270, YZU 171499, YZU 181050 and YZU 181280 fell into four different subclades of *A. alternata*. Strains of YZU 161051, YZU 161186, YZU 161119 and YZU 191090 were grouped well together with *A. iridicola* supported by values of 1.0/100% (PP/BS). Two strains of YZU 191101 and YZU 191076 fell into a distinct single lineage (PP/BS = 1.0/99%) close to *A. gaisen* supported by the values of 1.0/100% (PP/BS), which could be considered as a new species. The remaining two strains of YZU 161050 and YZU 161052 also formed an individual branch with high PP/BS values of 1.0/100% representing a new species in the phylogram, sister to *A. iridicola* (PP/BS = 0.99/98%; Figure 2). Besides, the four strains, *A. gaisen* and *A. iridicola* were grouped together supported with medium PP/BS values of 0.8/65%.

### Morphology

**Figure 3:**
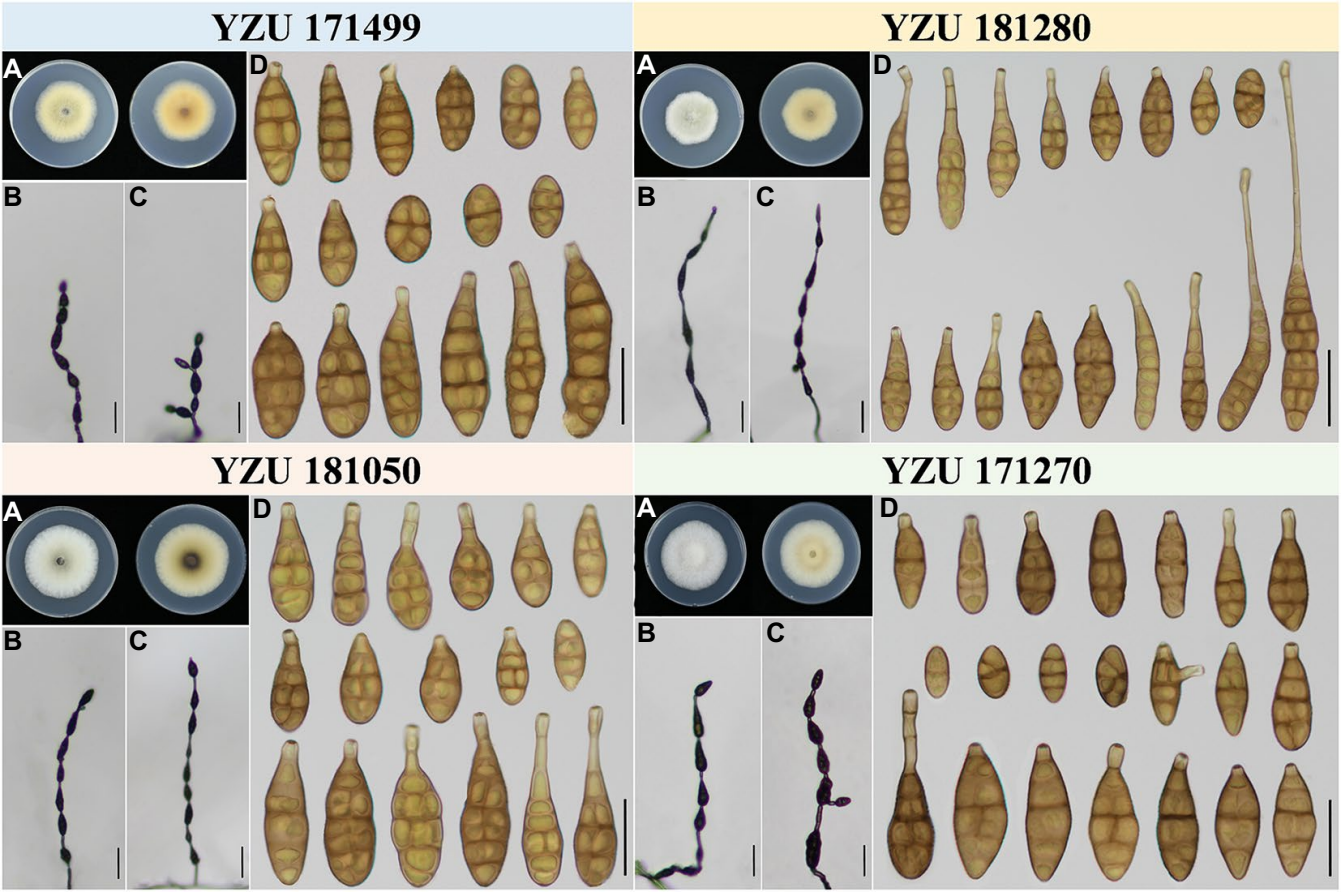
Morphology of the represent four strains of *A. alternata.* **(A)** Colony phenotype (on PDA for 7 days at 25°C); **(B,C)** Sporulation patterns (on PCA at 22°C); **(D)** Conidia Bars: **B,C** = 50 μm; **D** = 25 μm.

**Figure 4:**
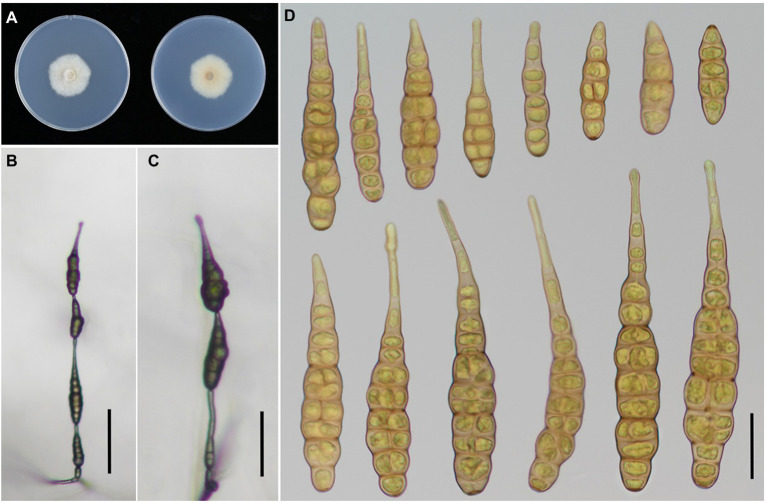
Morphology of *Alternaria iridicola.* **(A**) Colony phenotype (on PDA for 7 days at 25°C); **(B,C)** Sporulation patterns (on PCA at 22°C); **(D)** Conidia Bars: **B,C** = 50 μm; **D** = 25 μm.

**Figure 5:**
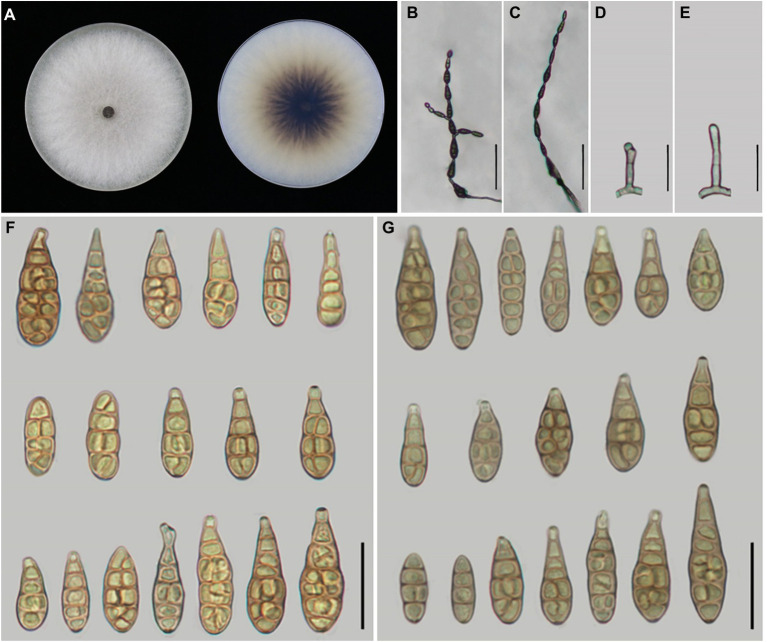
Morphology of *Alternaria setosae* sp. nov. **(A)** Colony phenotype (on PDA for 7 days at 25°C); **(B,C)** Sporulation patterns (on PCA at 22°C); **(D,E)** Conidiophores; **(F)** Conidia (on PCA at 22°C); **(G)** Conidia (on V8A at 22°C). Bars: **B,C** = 50 μm; **D–G** = 25 μm.

**Figure 6:**
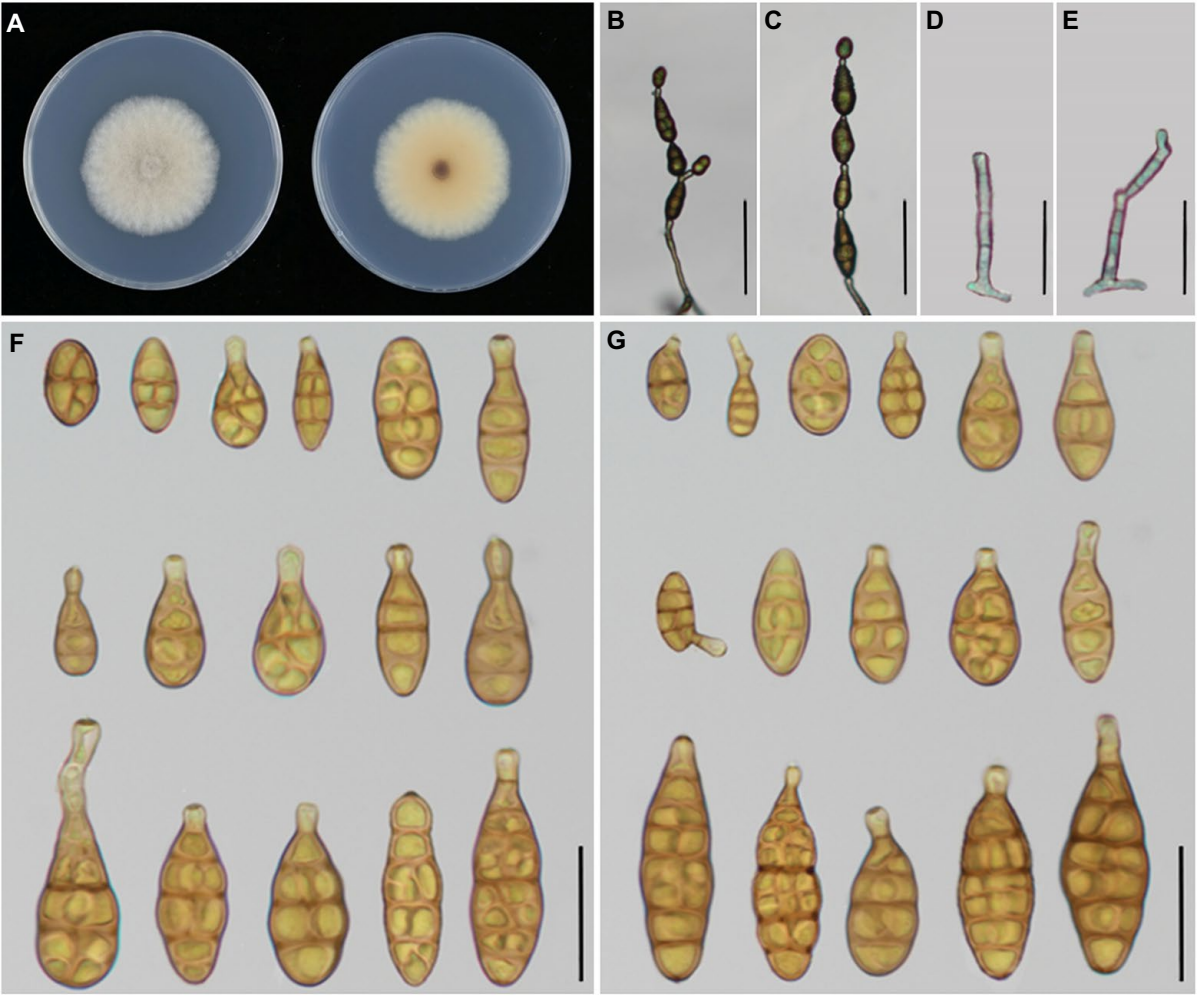
Morphology of *Alternaria tectorum* sp. nov. **(A)** Colony phenotype (on PDA for 7 days at 25°C); **(B,C)** Sporulation patterns (on PCA at 22°C); **(D,E)** Conidiophores; **(F)** Conidia (on PCA at 22°C); **(G)** Conidia (on V8A at 22°C). Bars: **B,C** = 50 μm; **D–G** = 25 μm.

According to morphological traits, the present *Alternaria* strains associated with *Iris* spp. were *A. alternata* (Figure 3) and *A. iridicola* (Figure 4) and two new species of sect. *Alternaria* (Figures 5, 6; Table 3). The two new species were illustrated and described in this study.

**Table 3:** Morphological comparison of the present *Alternaria* and their relevant species.

Species/Strain	Conidia				Conidia per chain	References
	Shape	Body (μm)	Beak (μm)	Septa		
*A. alternata*	Ovoid, ellipsoid, or subsphaeroid or with short beak	7–30 (−40) × 5–12	3–5 (−30)	1–7	4–20	Simmons (2007)
**YZU 171270**	**Almost slender ellipsoid or ovoid, sometimes with short beak**	**10–36 × 6–23**	**4–17.5**	**1–5**	**3–8**	**This study**
**YZU 171499**	**Ovoid or ellipsoid with cylindric apex**	**14–51 (−66.5) × 7.5–18.5**	**–**	**1–6**	**4–8**	**This study**
**YZU 181050**	**Ovoid, or ellipsoid or with blunted beak**	**11.5–40 × 4–7.5**	**5.5–26**	**3–6**	**5–10**	**This study**
**YZU 181280**	**Ellipsoid, ovoid, almost with long beak**	**22–76.5 (−84.5) × 7.5–16**	**6–67.5 (–71.5)**	**1–9**	**3–8**	**This study**
*A. gaisen*	Short to long ovoid or ellipsoid	30–45 (−55) × 13–15 (−18)	–	5–8	3–9	Simmons (2007)
	Ovoid or ellipsoid	20–55 × 10–15	–	3–7	3–10	Yu (2015)
*A. iridiaustalis*	Broad-ovoid or broad-ellipsoid and long ellipsoid	30–50 × 13–24	–	3–4	1–2	Simmons (2007)
	Ellipsoid or long ellipsoid	20–50 × 15–24	15–100 (−133)	3–5	1–2	Luo et al. (2018)
*A. iridicola*	Broadly obclavate with a blunt conical or sturdy cylindric apex	60–125 × 17–33	20**–**70	5–9	3–9	Simmons (2007)
	**Obclavate or long ovoid, sometimes cylindrical with columnar beak**	**36–74 × 3–26**	**10–74**	**1–8**	**4–5**	**This study**
	Obclavate or long ovoid with columnar beak sometimes	21–127 × 7–38	–	2–16	3–4	Nishikawa and Nakashima (2020)
***A. setosae* sp. nov.**	**Short to long ovoid or ellipsoid**	**(12–) 14–45 × 7–14**	**–**	**1–6**	**4–12**	**This study**
***A. tectorum* sp. nov**.	**Broad ovoid or broad ellipsoid**	**13–38.5 × 5.5–13**	**–**	**1–6**	**2–9**	**This study**

### Taxonomy

***Alternaria setosae* Y.N. GOU & J.X. Deng, sp. nov**. Figure 5

**MycoBank No.: 845326**

**Etymology:** In reference to the pathogenic host species name, *Iris setosa*.

**Typifification:** China, Fujian Province, Fuzhou City, from leaf spot of *Iris japonica*, 15 April 2019, J.X. Deng, (YZU-H^−0046^, holotype), ex-type culture YZU 191101.

**Description:** Colonies on PDA circular, light cottony and white to off-white in the center, villiform with white at the edge, reverse dark brown at centers, with scattering shape, 83.4–84.8 mm in diam., at 25°C for 7 days. On PCA, conidiophores arising from substrate or lateral of aerial hyphae, straight or curved, smooth-walled, septate, pale brown; (12.5–)15–40 × 3–5 μm (av.: 24.5 × 4 μm); conidia 4–12 units per chain, medium yellow-brown with almost smooth-walled, short to long-ovoid or ellipsoid, (12–)14–45 × 7–14 μm (av.: 31.5 × 10.5 μm)，1–6 transverse septa，0–2(−3) longitudinal septa. On V8A, conidiophores straight or curved, smooth-walled, septate, 18–43(−50.5) × 3.5–5 μm (av.: 31 × 4 μm), conidia 4–11 units in a chain, medium yellow-brown, almost smooth to conspicuously elliptoid overall, 20–43 × 7–12.5 μm (av.: 30 × 9.5 μm), 2–7 transverse septa, 0–2(−3) longitudinal septa.

**Notes:** Phylogenetic analysis reveals that the species is sister to *A. gaisen* on the basis of a combined dataset of ITS, *GAPDH*, *TEF1*, *RPB2*, *Alt a 1*, OPA10-2 and *EndoPG* gene regions. After a nucleotide pairwise comparison with *A. gaisen* in those seven regions, there are 1/470 bp, 1/534 bp, 3/240 bp, 3/603 bp, 0/429 bp, 16/622 bp and 0/442 bp site differences, respectively. Morphologically, this species can be differentiated by producing smaller conidia with less transverse septa (Table 3). It also readily distinct to *A. tectorum* sp. nov. and *A. iridicola* in sporulation pattern and conidial size (Table 3).

***Alternaria tectorum* Y.N. GOU & J.X. Deng, sp. nov**. Figure 6

**MycoBank No.: 845325**

**Etymology:** In reference to the host species name, *Iris tectorum*.

**Typifification:** China, Hubei Province, Jingzhou City, from leaf spot of *Iris tectorum*, 21 May 2016, J.X. Deng, (YZU-H^−0025^, holotype), ex-type culture YZU 161050.

**Description:** Colonies on PDA circular, cottony with dense hyphae, off-white, reverse buff in the center, with white margin, 50–52 mm in diam., at 25°C for 7 days. On PCA, conidiophores arising from substrate, straight to slightly curved, septate, pale to dark brown, 32–58.5(−62.5) × 3.5–4.8 μm (av.: 45.5 × 4 μm); conidia 1–6 in a chain, solitary or straight in chains with few branches, broad-ovoid or broad-ellipsoid, smooth-walled, 13–38.5 × 5.5–13 μm (av.: 23.5 × 9 μm), 0–6 transverse septa, 0–2(−3) longitudinal septa, On V8A, conidiophores straight to slightly curved, septate, pale to dark brown, 31.5–54.5 × 3.5–4.75 μm (av.: 41.5 × 4 μm)，conidia 2–7 per chain, pale to medium yellowish brown, smooth walled, broad-ovoid or broad-ellipsoid, 13.5–37.5 × 5.5–13.5 (av.: 26 × 9.5 μm), 1–7 transverse septa, 0–2(−3) longitudinal septa.

**Notes:** Phylogenetic analysis shows that the species (13 –38.5 × 5.5 –13 μm in body size) falls in an individual clade with high PP/BS values of 1.0/100% representing a new species in the phylogram, sister to *A. iridicola* (47 -87 × 15 -27 μm in size), but it can be significantly distinguished by the conidial size and shape (Table 3). Comparing with *A. iridicola,* the present species contains 1/442 bp, 16/603 bp and 37/622 bp nucleotide differences in *EndoPG*, *RPB2* and OPA10-2 gene sequences, respectively.

### Pathogenicity tests

**Figure 7:**
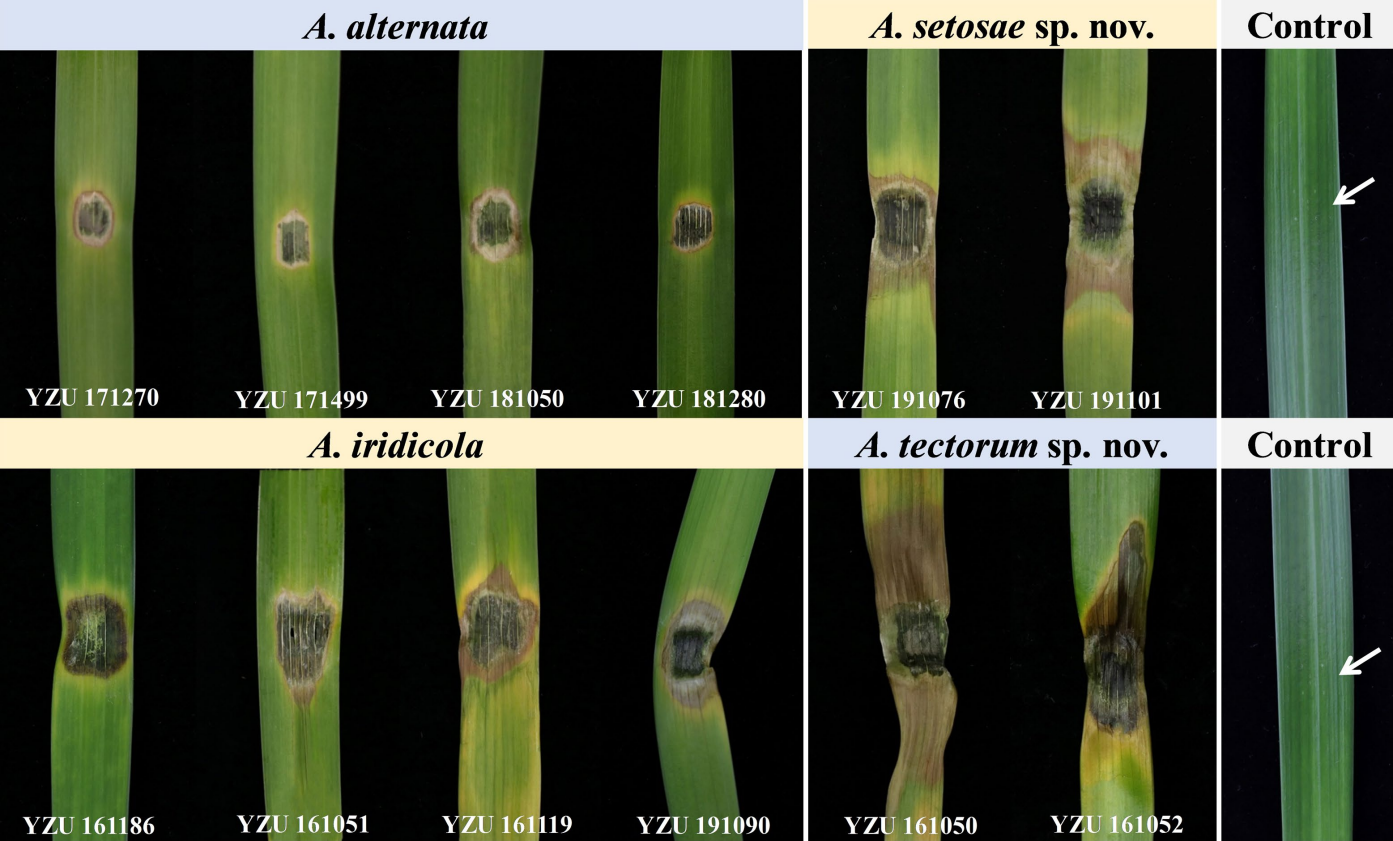
Pathogenicity of the present *Alternaria* species on *Iris setosa*.

Pathogenicity tests inoculated with those 12 *Alternaria* strains for 7 days, the results showed that all strains were 100% pathogenic to *Iris setosa* (Table 4; Figure 7). When inoculated with mycelium discs, the small spot was normally appeared after 4 days on the living leaves. Then it expanded into nearly round to long elliptic, dark brown necrotic lesions coupling with a narrow yellow halo around the periphery of the spot. Besides, the necrotic part of spot is easy to rupture and form perforation. For some strains (*A. tectorum*), the spots spread quickly until most of the leaves becoming withered causing blight. The symptoms were identical to those in the fields (Figure 1). The virulence among the species was variable (Table 4). Strains of *A. tectorum* sp. nov. showed the most virulent to *I. setosa* with the lesion size (LS) around 48 to 49 mm, followed by *A. setosae* sp. nov. (LS around 26–27), then *A. iridicola* and *A. alternata*. There were no symptoms on the control leaves. To verify Koch’s rules, the re-isolation of the causal pathogen was performed, which was consistent with the inoculated strains.

**Table 4:** Disease incidence and lesion size on *Iris setosa* induced by the present *Alternaria* species.

Species	Strain	Disease incidence (%)	Lesion (mm)
*A. tectorum* sp. nov.	YZU 161050	100 ± 0 a	48.97 ± 0.65 a
	YZU 161052	100 ± 0 a	48.28 ± 0.36 a
*A. setosae* sp. nov.	YZU 191101	100 ± 0 a	26.87 ± 0.31 b
	YZU 191076	100 ± 0 a	26.76 ± 0.25 b
*A. iridicola*	YZU 161051	100 ± 0 a	24.15 ± 0.33 bc
	YZU 191090	100 ± 0 a	23.55 ± 0.22 bc
	YZU 161119	100 ± 0 a	22.48 ± 0.54 c
	YZU 161186	100 ± 0 a	22.51 ± 0.60 c
*A. alternata*	YZU 171270	100 ± 0 a	12.39 ± 0.63 d
	YZU 181050	100 ± 0 a	11.77 ± 0.59 d
	YZU 181280	100 ± 0 a	10.22 ± 0.40 d
	YZU 171499	100 ± 0 a	10.08 ± 0.28 d

*Disease incidence (DI) was evaluated by counting the percentage of diseased leaves*. Lesion size (LS = the maximum lesion length) values are the mean value of three replicates ± standard deviation. Values followed by different lowercase letters within a column are significantly different according to the least significant difference test (P < 0.05) using IBM SPSS Statistics 23.

## Discussion

The sect. *Alternaria* is found containing 17 species and a species complex of *A. arborescens* (AASC; Woudenberg et al., 2015; Li et al., 2022). After a large-scale sample collection of *Iris* plants in China, two known species (*A. alternata* and *A. iridicola*) and two novel species in sect. *Alternaria* were detected in this study. In addition, the two new species also identified and described as *A. setosae* sp. nov. and *A. tectorum* sp. nov. based on the morphological characteristics and multi-locus phylogenetic analyses.

*Alternaria alternata* is one kind of small-spored *Alternaria*, which taxonomy is controversial due to the similar conidial morphology (Zhang, 2003; Simmons, 2007). Later, it is classified as one species based on multi-gene phylogenetic analyses, encompassing 35 morphospecies identified by Simmons (2007) which could not reliably distinguish those species (Woudenberg et al., 2015). Meanwhile, it resulted in confusion to the related taxonomic works on their classification. Morphologically, strain YZU 171270 grouped in a sub-clade containing the type strain of *A. alternata* (Figure 2) can form long catenulate chain with branching 1 to 3, while strain YZU 181050 in its sister sub-clade containing the type strain of *A. tenuissima* can sporulate with long straight conidial chains without branch. The results agreed with Simmons (2007) for the descriptions of *A. alternata* and *A. tenuissima*. In China, *A. tenuissima* has been reported on *I. tectorum* in Qingdao city (Sun et al., 2019) and *I. ensata* in Anqing city (Li et al., 2020), whose sporulation patterns are not provided. Although sporulation phenotype cannot accurately reflect the evolutionary relationship, it still is of great value for subgroup classification of *A. alternata*. Besides, the present strains falling into different clade or subclade (Figure 2) are highly variable (Figure 3). For example, both strains YZU 181050 and YZU 181280 are similar in sporulation, but strain YZU 181280 is characterized by comprising larger conidia with blunted beak up to 71.5 μm long (Figure 3). The morphological boundary of *A. alternata* has not been clearly defined yet and how to classify them accurately needs further study (Aung et al., 2020).

*Alternaria iridicola* has been recognized as a large-spored *Alternaria* (*ca.* 60–125 × 17–33 μm in size) comprising broadly obclavate conidia with a blunt conical or sturdy cylindric apex, which is a host specific species to *Iris* plants (Simmons, 2007). Gannibal and Lawrence (2018) considered that *A. iridicola* shares traits with both sect. *Panax* and *Porri* of *Alternaria*, which was not easily distinguished in terms of section affiliation. Latterly, this species is the only large-spored *Alternaria* in small-spored sect. *Alternaria* according to multi-locus phylogenetic analysis (Nishikawa and Nakashima, 2020). Nishikawa and Nakashima (2020) took experiments to assess the host range of *A. iridicola*, for which *I. laevigata* and *I. hollandica* were proved host related, except *I*. *ensata*. The species was firstly reported causing leaf spot disease on *I. tectorum* from Xinxiang city of China in 2015 (Zhai et al., 2015). In this study, *I. japonica* is a new host for *A. iridicola* in China, which also can infect *I. setosa*.

In this study, two new species of *A. setosae* and *A. tectorum* are grouped with *A. gaisen* and *A. iridicola* in a clade, sister to *A. alstroemeriae*. The morphological comparisons are listed in Table 3. Compared with *A. gaisen* (30–45 (−55) × 13–15 (−18) μm with 5–8 septa), *A. setosae* sp. nov. is different by producing smaller conidia (14–45 × 7–14 μm) and less transverse septa (1–6; Simmons, 2007). In comparison with *A. iridicola*, *A. tectorum* sp. nov. is an obviously a small-spored species (Simmons, 2007). The results increase two members for sect. *Alternaria*. In the previous reports, *A. iridiaustralis* has been reported on *Iris* spp. in Australia and New Zealand (Simmons, 2007) and on *Iris ensata* as causal foliar pathogen in China (Luo et al., 2018), which is characterized by broad-ovoid or broad-ellipsoid and long ellipsoid conidia, obviously not like the present four species.

During the isolation of *Alternaria* from *Iris* plants, *A. alternata* like species are frequently observed on moisten cultured leaf tissues, such as *A. alternata*, *A. iridicola*, *A. setosae* sp. nov. and *A. tectorum* sp. nov.. Consequently, their virulence is assessed on wounded leaves of *I. setosa*, and considerable variation is observed among the species in the pathogenicity tests, from which *A. tectorum* sp. nov. shows the most pathogenic, followed by *A. setosae* sp. nov., *A. iridicola*, and finally *A. alternata*. The pathogenic mechanism comprises mechanical penetration, secreting degradation enzymes, metabolites, and toxins, which causes plant diseases (Meng et al., 2009). *Iris* plants are near to the ground growing, which suffers vulnerable damage caused by raining splashing, animals, and so on. When there are small wounds on *Iris* leaves, it is easier to attack by pathogens, which could significantly lower their economic and ornamental values. On the other hand, mycelia discs of the four species were inoculated on unwounded living leaves of *Iris setosa* for 14 days (data not shown). *Alternaria tectorum* sp. nov. (LS around 20 mm) and *A. iridicola* (LS 6 to 18 mm) can penetrate the leaves and induce symptoms. While *A. alternata* and *A. setosae* sp. nov. fail to penetrate and cause infection. The present results will provide experimental evidence and reference for the prevention and control of *Iris* leaf spot caused by *Alternaria alternata* like species.

## Data availability statement

The datasets presented in this study can be found in online repositories. The names of the repository/repositories and accession number(s) can be found in the article/supplementary material.

## Author contributions

JD, SA, and YG did the study design. JD, SA, CH, and AH collected the diseased leaf samples. YG and SA performed the experiments and analyzed the data. YG wrote the manuscript. JD and YG contributed to manuscript revision, read, and approved the submitted version.

## Funding

This study was financed by the National Natural Science Foundation of China (31400014 and 32270022).

## Conflict of interest

The authors declare that the research was conducted in the absence of any commercial or financial relationships that could be construed as a potential conflict of interest.

## Publisher’s note

All claims expressed in this article are solely those of the authors and do not necessarily represent those of their affiliated organizations, or those of the publisher, the editors and the reviewers. Any product that may be evaluated in this article, or claim that may be made by its manufacturer, is not guaranteed or endorsed by the publisher.

## Footnotes

^1^ https://www.ncbi.nlm.nih.gov/

^2^ www.ncbi.nlm.nih.gov
